# Perceived Social Norms as Determinants of Adherence to Public Health Measures Related to COVID-19 in Bali, Indonesia

**DOI:** 10.3389/fpubh.2021.646764

**Published:** 2021-04-30

**Authors:** Putu Ayu Indrayathi, Pande Putu Januraga, Putu Erma Pradnyani, Hailay Abrha Gesesew, Paul Russel Ward

**Affiliations:** ^1^Department of Public Health and Preventive Medicine, Faculty of Medicine, Udayana University, Bali, Indonesia; ^2^Center for Public Health Innovation, Faculty of Medicine, Udayana University, Bali, Indonesia; ^3^College of Medicine and Public Health, Flinders University, Adelaide, SA, Australia; ^4^Flinders Health and Medical Research Institute, Flinders University, Adelaide, SA, Australia; ^5^Epidemiology, School of Health Sciences, Mekelle University, Mekelle, Ethiopia

**Keywords:** COVID-19, social norm, prevention protocol, adherence, online survey

## Abstract

**Introduction:** Before the widespread availability of an effective COVID-19 vaccine, it is crucial to control the rate of transmission by ensuring adherence to behavioral modifications, such as wearing masks, physical distancing, and washing hands, all of which can be implemented as public health measures. Focusing on the conditions in Bali, this study explored the level of compliance to public health measures targeted at COVID-19 and identified the determinants of compliance via the values, rules, and knowledge approach.

**Materials and Methods:** This cross-sectional study conducted an online survey using the Google Form application from June 29 to July 5, 2020. The minimum required sample size was 664. Inclusion criteria were set as follows: 18 years of age or older and residing in Bali during the data collection period. Adherence was measured based on nine protocol indicators that were rated using a four-point Likert scale. A multiple linear regression analysis was then conducted to determine the associated factors of adherence to public health measures.

**Results:** Of the 954 survey respondents, data from 743 were included for analysis. The average level of adherence to public health measures was 32.59 (range of 20–36). The linear regression analysis showed that perceived health benefits from public health measures, being female, and having COVID-19 test histories were significantly associated with adherence to public health measures.

**Conclusions:** For public health measures targeted at COVID-19, adherence was strongly associated with perceived social norms, in which individuals played social community roles by adapting to standardized public health measures. It is thus imperative for governments to support and monitor public health measures during the COVID-19 pandemic.

## Introduction

Coronavirus disease (COVID-19) was first identified in Wuhan, China, on December 31, 2019. Due to the ease of transmission, it is now found across the globe. In fact, ~71.4 million confirmed cases and 1.6 million deaths were attributed to COVID-19 as of December 13, 2020 (1). The pandemic has also caused a variety of social, political, and economic crises, some of which have resulted from the unintentional impacts of public health measures targeted at controlling the virus (2). The development of COVID-19 is still fluctuating in Indonesia. As of now, it is unclear whether the country has seen the peak of the pandemic, with the number of confirmed cases reaching 605,000 on December 13, 2020, including 18,511 deaths (3).

The World Health Organization (WHO) has suggested several public health measures for containing viral transmission. In this context, Indonesian policies for handling COVID-19 are implemented through health promotions, particularly those involving the implementation of clean and healthy lifestyles, social and physical distancing, mandates for studying and working at home, universal mask usage, screening, and large-scale social restrictions in areas that are undergoing significant increases in the number of cases (4–6). Although the COVID-19 pandemic has not yet peaked, the Indonesian nation plans to officially adapt to “the new normal” by the end of May. According to the Indonesian Department of Health, “the new normal” is defined as the widespread implementation of productive and safe community activities that adhere to COVID-19 prevention measures, including mandatory mask usage, safe social distancing, the practice of always washing hands with soap and running water, regular exercise, adequate rest, the avoidance of panic, and nutritious diets (7). These adaptations were initially made based on epidemiological indicators of the reproduction number (R0) associated with the pandemic and limited to certain sectors (8). While most regions in Indonesia have officially declared their intent to adapt to the new normal, many locations have not yet met the epidemiological indicators; some have even seen increases in the number of cases.

Various factors are required to ensure an effective adaptation process, which is facilitated when individuals have sufficient knowledge about newly introduced habits. Research related to community knowledge, attitudes, and behavior toward social distancing policy as a means of preventing transmission of COVID-19 in Indonesia showed that 99, 59, and 93% of respondents have good knowledge, positive attitudes, and good behavior toward social distancing, respectively. Among the respondents who had good knowledge, 58.85% showed positive attitudes, and 93.3% have good behavior. The vast majority of the respondents who had positive attitudes showed good behavior (96.7%) (9). The knowledge, attitudes, and practices of using masks by the community are efficient in the prevention of the spread of COVID-19 infection (10). Knowledge, attitudes, and practices (KAP) toward COVID-19 play pivotal roles in assessing the willingness of a community to adopt behavioral change initiatives during the pandemic (11). This is associated with a better understanding of the related values and benefits, which are supported by regulations that encourage the implementation of both formal and informal behaviors (e.g., social pressures resulting from observed behaviors or adaptations made by others) (12, 13). This study examined the level of adherence to these types of adaptations in Bali, specifically those targeted at achieving the new normal through COVID-19 prevention measures. This investigation was accomplished via the values, rules, and knowledge (VRK) approach, which can later be structured into an improved adaptation strategy.

## Materials and Methods

### Study Design and Setting

This study conducted an online cross-sectional survey using the Google Form application from June 29 to July 5, 2020. The required sample size was calculated using the survey formula, based on the 99% confidence interval (CI), 0.50 proportion of adherence, and 0.05 precision, the minimum required size was 664 respondents. This study used the 99% confidence level in the sample calculation to increase the chances of getting a reliable sample of the population parameters if the estimation process is carried out repeatedly and minimizes the risk of error results obtained from the sample. We invited respondents through a Google link form listed on a poster showing information about the study purpose, which was shared via both Facebook and WhatsApp. Poster then shared through researcher's networking and social media influencers. Eligibility criteria were set as follows: aged 18 years or older, residing in Bali during the data collection period, and willing to participate. Those who did not meet the eligibility criteria and submitted incomplete answers were excluded from the study. During the data collection period, the Balinese government relaxed its COVID-19 restrictions on community activities between districts and cities. A special task force was also established to address the spread of COVID-19 through traditional markets.

Bali is among the top 10 Indonesian provinces showing the highest number of COVID-19 cases, with ~14,596 cases and 476 deaths recorded as of December 17, 2020 (14). In congruence with the central government campaign, Bali implemented new customs and adaptations on July 5, 2020 (15, 16). As it does not seem likely that local governments will directly limit personal activities, COVID-19 control measures will heavily depend on whether individual communities can adapt to these new habits, especially those which are part of the prevention protocol (16, 17). As such, the capacity to adapt to these public health measures is an essential factor for success, particularly while there is no widely available vaccine for containing or preventing COVID-19.

### Study Variables

In this study, the dependent variable was set as “adherence to COVID-19 public health measures,” which was determined based on the total score achieved after combining scores from individual indicators, including mask usage, hand washing, keeping distance, changing clothes, covering the nose when sneezing/coughing, and avoiding crowds. Each item was rated on a Likert scale ranging from 1 (never) to 4 (always).

The independent variables consisted of the following five main factors:

- Demographic characteristics (i.e., gender, age, marital status, number of children, education level, district of residence, and type of occupation). Education level was divided into primary education (did not attend formal school, through high school) and university (diplomas to graduate school). Marital status and number of children were combined to form the following categories: unmarried, married with no children, and married with children. Finally, type of occupation included civil servants, private employees/laborers, others (freelancers, self-employed, farmers, and traders), unemployed, and students.
- Perception of the value of public health measures (i.e., value of health, economic, and social benefits). This variable was assessed based on the total scores achieved after combining scores from responses to the health-related benefits of mask usage, washing hands, and keeping distance. Each statement was rated according to a Likert scale ranging from 1 (strongly disagree) to 4 (strongly agree). An item was measured based on health, e.g., “The health protocol for wearing a mask during activities has benefited me in maintaining my health.” Perception of the value of economic benefits was assessed based on the total scores achieved after combining scores from responses to the economic/job benefits of mask usage, washing hands, and keeping distance. Here, each item was also rated according to a Likert scale ranging from 1 (strongly disagree) to 4 (strongly agree). An item was measured based on the value of economic benefits, e.g., “The health protocol for wearing a mask helps my business and my job.” Perception of the value of social benefits was measured based on responses to one item, which was scored according to the same Likert scale ranging from 1 (strongly disagree) to 4 (strongly agree). Perception of the value of social benefits was measured based on “The health protocols for wearing a mask, washing hands, and physical distancing have disturbed my social life.”
- Perception of the rules for COVID-19 control (i.e., social norms and formal rules). Social norms were assessed based on the total scores achieved after combining scores from six items related to community participation; that is, whether people in the community implemented public health measures. All items were measured according to a Likert scale ranging from 1 (never) to 4 (always). Item was measured based on social norms, e.g., “People in my area keep their distance and reduce physical contact.” Formal rules were assessed based on the total scores achieved after combining scores from five items related to formal government regulations targeted at public health measures. All items were measured using a Likert scale ranging from 1 (never) to 4 (always). Item was measured based on formal rules, e.g., “The current regulations for COVID-19 require masks when performing activities outside and working.”
- Knowledge of COVID-19 and public health measures targeted at the new normal were assessed based on the total scores achieved after combining scores from 10 items that were answered and scored as follows: correct answer choice (1) and wrong/do not know (0) for positive questions, and wrong answer choice (1) and true/do not know (0) for negative questions. Item was measured for knowledge of COVID-19, e.g., “The main clinical symptoms of COVID-19 are fever, dry cough, sore throat, loss of smell, and breathing difficulties.”
- Other factors related to health protocol adherence, including risk perception, fear perception, trust in the government, COVID-19 test history, respondent health status, and access to COVID-19 prevention instruments. Risk perception was measured based on one statement that was answered according to a Likert scale ranging from 1 (strongly disagree) to 5 (strongly agree). Fear perception was measured based on the total scores achieved after combining scores for seven items, each of which were answered according to a Likert scale ranging from 1 (strongly disagree) to 5 (strongly agree) (17). History of COVID-19 tests (either via swab PCR or rapid testing) was answered as either No or Yes. Trust in the government was assessed based on one statement, which was answered according a Likert scale ranging from 1 (disagree) to 4 (strongly agree). Individual health status was measured according to a Likert scale ranging from 1 (very bad) to 5 (very good), then divided for analysis purposes into categories of bad (very bad to sufficient), good, and very good. Access to COVID-19 prevention instruments was assessed based on four items related to individual access to masks and handwashing locations as well as whether participants were accustomed to washing their hands and using hand sanitizer; all factors were rated according to a Likert scale ranging from 1 (strongly agree) to 4 (strongly disagree).

Testing the validity and reliability of the instrument with related experts was carried out before the instrument was disseminated, and to strengthen this, the validity and reliability tests were carried out simultaneously following the research data collection. From a total sample of 743, the results of validity and reliability tests with the Pearson correlation statistical test (*r* count > *r* table or ir-cor more than 0.3) and Cronbach alpha (>0.6) meaning that the instrument used is valid and reliable.

### Statistical Analysis

All data were edited and cleaned for analysis. Descriptive statistics were used to obtain variable distributions (i.e., frequencies, percentages, means, and standard deviations). We applied a bivariate linear regression test to determine crude associations between independent and dependent variables; we nominated candidate variables with *p*-values < 0.25. A multiple linear regression analysis was performed to determine which independent variables were associated with the dependent variable. Results were considered significant based on *p*-values < 0.05. All data analyses were conducted using Stata 14.0.

### Ethical Approval

This study received approval with Ethics Decree Number: 1303 /UN14.2.2.VII.14/LT/2020, dated June 23, 2020, from the Ethics Commission, Faculty of Medicine, Udayana University. All respondents gave their consent to participate. The first 500 were rewarded with telephone credits or electronic money transfers amounting to Rp 25,000 (around USD $1.78), while eight participants were randomly selected to received amounts of Rp 250,000 (around USD $17.76).

## Results

We initially received a total of 954 individual survey responses, but only 743 (77.8%) of these were analyzed (i.e., participants met the eligibility criteria and submitted complete answers). Table 1 shows their sociodemographic characteristics. As shown, most respondents were women (63%), relatively young (88% were <40 years of age), and unmarried (52%). Furthermore, a large majority (74%) were university graduates. Finally, most (36%) worked in the private sector.

**Table 1:** Sociodemographic characteristics of the study participants.

**Variable**	***n* (%)**
**Sex**	
Male	274 (36.88)
Female	469 (63.12)
**Age**	
<20 years	63 (8.48)
21–30 years	433 (58.28)
31–40 years	161 (21.67)
>40 years	86 (11.57)
**Marital status and number of children**	
Single	383 (51.55)
Married without children	62 (8.34)
Married with children	298 (40.11)
**Latest education**	
Primary education	194 (26.11)
University	549 (73.89)
**Districts**	
Outside Denpasar	445 (59.89)
Denpasar	298 (40.11)
**Type of occupation**	
Student	120 (16.15)
Not working	87 (11.71)
Others (self-employed, farmer, trader)	142 (19.11)
Private employee/laborer	268 (36.07)
Civil servant	126 (16.96)

Table 2 shows the distribution of indicators for COVID-19-related public health measures. As shown, average adherence was 32.59 (range of 20–36). Detailed information on the adherence levels per question item are available in Supplementary File 2. In summary, the highest scores were found for compliance with mask usage when going outdoors, while the lowest were found for doing so with the family when at home. The perceptions of health and economic value related to the application of public health measures produced averages of 10.82 and 10.62, respectively (total scores ranging from 5 to −12). The proportions of answers per item for the perceptions of health and economic value are available in Supplementary File 3. Results also showed that a high proportion of respondents expressed disapproval due to health protocols that interfered with their social lives. For the perception of rules, the average social norm score was 19.09 out of a possible total score ranging from 6 to 24, while the average formal rule score was 16.84 out of a possible total score ranging from 5 to 20. The complete results for the perceptions of formal rules and social norms are available in Supplementary File 4. Other factors concerning knowledge related to COVID-19 obtained an average of 9.17 out of a possible total score ranging from 0 to 10. The complete results for the knowledge section are available in Supplementary File 5. Survey results also showed an average score for COVID-19 fear perception of 20.50 out of a total possible score ranging from 7 to 35. A similar trend was found for perceived risk, in which only about half of the respondents agreed or strongly agreed that there was a high risk of COVID-19 infection. Access to COVID-19 prevention instruments showed an average of 13.16 out of a total possible score ranging from 7 to 16. Detailed results for both these variables are available in Supplementary Files 6, 7, respectively. Finally, a high proportion of respondents trusted the government to control the spread of COVID-19.

**Table 2:** Distribution compliance to COVID-19 prevention measures and VRK constructs.

**Variable**	***n* (%)**
Public health measures compliance (mean ± SD)	32.59 (3.08)
Perception of the value of health benefits (mean ± SD)	10.82 (1.36)
Perception of the value of economic benefits (mean ± SD)	10.62 (1.49)
**Perception of social value (public health measures affect social life)**	
Strongly agree	85 (11.44)
Agree	132 (17.77)
Disagree	428 (57.60)
Strongly disagree	98 (13.19)
Perception of social norms (mean ± SD)	19.09 (3.43)
Perception of formal rules (mean ± SD)	16.84 (2.59)
Knowledge (mean ± SD)	9.17 (1.61)
**COVID-19 test history**	
No	558 (75.10)
Yes	185 (24.90)
**Health status**	
Bad	41 (5.52)
Good	368 (49.53)
Very good	334 (44.95)
**Perception of risk**	
Strongly agree	106 (14.27)
Disagree	84 (11.31)
Neutral	190 (25.57)
Agree	281 (37.82)
Strongly disagree	82 (11.04)
Perception of fear (mean ± SD)	20.50 (5.31)
Access to masks and washing hands (mean ± SD)	13.16 (1.78)
**Trust in the government**	
Disagree	47 (6.33)
Neutral	122 (16.42)
Agree	324 (43.61)
Strongly agree	250 (33.65)

Table 3 shows the results of both the bivariate and multiple linear regression analyses. The bivariate analysis showed that several factors were statistically associated with adherence to public health measures, including being female, married with children, COVID-19 test histories, health status, risk perception, fear perception, access to masks and handwashing, trust, valuing public health measures, and perceived social norms and rules. However, the multiple linear regression analysis only showed that perceived social norms, perceived health benefits from public health measures, being female, and COVID-19 test histories were significantly associated with adherence to public health measures.

**Table 3:** Factors affecting adherence to public health measures.

**Variable**	**Bivariate analysis**				**Multivariate analysis**			
		**95% CI**		***p*_value**		**95% CI**		***p*_value**
	**B**	**Lower**	**Upper**		**B**	**Lower**	**Upper**	
**Sex**								
Male								
Female	0.96	0.51	1.42	0.000^*^	0.58	0.18	0.98	0.004^*^
**Age**								
<20 years								
21–30 years	0.46	−0.35	1.27	0.264				
31–40 years	0.29	−0.61	1.19	0.531				
>40 years	0.36	−0.64	1.36	0.482				
**Marital status and number of children**								
Single								
Married without children	0.22	−0.61	1.04	0.587	−0.10	−0.84	0.63	0.781
Married with children	0.67	0.20	1.13	0.005^*^	0.18	−0.29	0.64	0.460
**Latest education**								
Primary education								
University	0.28	−0.22	0.79	0.270				
**Districts**								
Outside Denpasar								
Denpasar	0.03	−0.43	0.47	0.916				
**Type of occupation**								
Student	−0.54	−1.31	0.23	0.171	−0.32	−1.11	0.45	0.416
Not working	−0.72	−1.55	0.12	0.092	−0.24	−1.03	0.53	0.539
Others (self-employed, farmer, trader)	0.14	−0.59	0.88	0.704	0.23	−0.44	0.89	0.510
Private employee/laborer	0.31	−0.34	0.96	0.344	0.36	−0.23	0.95	0.236
Civil servant								
**COVID-19 test history**								
No								
Yes	1.02	0.51	1.52	0.000^*^	0.89	0.45	1.34	0.000^*^
**Health status**								
Bad								
Good	1.07	0.09	2.06	0.032^*^	0.59	−0.28	1.45	0.183
Very good	1.77	0.78	2.76	0.000^*^	0.81	−0.08	1.70	0.074
**Perception of risk**								
Strongly disagree								
Disagree	−0.82	−1.70	0.05	0.065	−0.07	−0.85	0.72	0.866
Neutral	−1.12	−1.84	−0.39	0.003^*^	−0.43	−1.18	0.23	0.197
Agree	−1.28	−1.96	−0.59	0.000^*^	−0.59	−1.36	0.07	0.078
Strongly disagree	−1.15	−2.03	−0.26	0.011^*^	−0.81	−1.66	0.02	0.056
Perception of fear	0.05	0.01	0.09	0.017^*^	0.03	−0.01	0.07	0.147
Access to masks and washing hands	0.39	0.27	0.51	0.000^*^	0.08	−0.04	0.22	0.184
Perception of the value of health benefits	0.58	0.43	0.74	0.000^*^	0.35	0.20	0.49	0.001^*^
Perception of the value of economic benefits	0.49	0.35	0.64	0.000^*^	0.06	−0.16	0.28	0.601
**Perception of the value of social benefits**								
Strongly agree								
Agree	−1.87	−2.70	−1.04	0.000^*^	−0.66	−1.44	0.13	0.099
Disagree	−0.77	−1.58	−0.17	0.015^*^	−0.18	−0.84	0.48	0.600
Strongly disagree	0.09	−0.97	0.79	0.841	−0.18	−0.96	0.62	0.661
Perception of social norms	0.41	0.35	0.46	0.000^*^	0.37	0.32	0.43	0.000^*^
Perception of formal rules	0.36	0.27	0.44	0.000^*^	0.03	−0.06	0.13	0.487
Knowledge	0.12	0.02	0.25	0.100	−0.02	−0.15	0.12	0.815
**Trust in the government**								
Disagree								
Neutral	−0.23	−1.25	0.79	0.656	−0.21	−1.13	0.71	0.657
Agree	0.78	−0.15	1.71	0.099	−0.07	−0.92	0.79	0.881
Strongly agree	1.38	0.43	2.32	0.004^*^	−0.50	−1.41	0.42	0.284

* *p ≤ 0.05*.

## Discussion

The COVID-19 pandemic has resulted in a variety of global changes that have impacted public health policies. Those designed to handle COVID-19 are typically conducted through health promotions targeted at the implementation of clean and healthy lifestyles, social and physical distancing, mandates to study and work at home, and mask usage during all community activities. This study found that the level of adherence to public health measures was directly proportional to social norms, meaning that individuals are more likely to adapt when those around them are also adaptive. In the COVID-19 era, social norms are more focused on the rules. Citizens are also expected to remind each other about the continual implementation of public health measures. The willingness and ability of community members to maintain behaviors that adhere to COVID-19 measures while advocating that others do so are crucial elements for widespread adoption (18). A social norm is what people in some groups believe to be normal in the group, that is, believed to be a typical action, an appropriate action, or both. It is believed that social norms may greatly influence health-related choice and behavior (19). In this context, the government must support formal rules and regulations while monitoring their implementation. Otherwise, unintentional consequences may arise, including, but not limited to, social interactional trouble between those who adopt and advocate public health measures and those who refuse to do so (18).

As described earlier, there are several health-related values and benefits to the practice of complying with public health measures (e.g., mask usage, washing hands with running water and soap, and keeping distances of 1–2 m). Mask usage can prevent the inhalation of large droplets and sprays but have limited ability to filter submicron-sized airborne particles of COVID-19 (20). Hand hygiene is essential for reducing COVID-19 transmission. Here are a variety of hand hygiene products available; however, their safety and efficacy vary (21). These elements increase personal safety while dramatically affecting adherence at the community level (22). Adaptation strategies can also be promoted through the consideration of existing social norms and increasing the overall perception of related benefits, particularly in terms of health. Policymakers and public health officers should be active in maximizing these benefits, which can increase the general level of social adherence to public health protocol (23).

Notably, this study also found that female respondents reported higher levels of adherence to public health measures than male respondents. This is similar to previous findings showing that women used masks and washed their hands at 12% higher rates (95% CI = 1.03–1.22, *p* < 0.05) than men (22). Moreover, females also declared a higher daily frequency of handwashing and washing their hands always when necessary more often than males. Males more often indicated various reasons for not handwashing, including there is no need to do it, they do not feel like doing it, and they have no time to do it (24). Based on other research, this may be rooted in the suggestion that women are generally more willing to maintain their health when compared with men (25).

In Bali, COVID-19 testing can be accomplished through either a rapid antibody test or PCR swab. In this study, respondents with histories of either type of COVID-19 test were more compliant than those who had never been tested. In addition, participation might have been higher among persons who knew someone who had tested positive or had died from COVID-19, which could have affected support for and adherence to mitigation efforts (26). Experiences with the COVID-19 test may also be related to higher perceived risks and perceived seriousness of the virus; in turn, this influences the decision to adopt public health measures targeted at COVID-19 prevention (27). Due to the low per-capita coverage rates of COVID-19 testing in Bali (and Indonesia as a whole), improved testing rates may therefore facilitate the adoption of public health measures designed to prevent transmission (28).

## Limitations

While this study produced valuable findings, there were also some limitations. Due to the nature of online surveys, respondent biases may have influenced the results. For example, low participation among the elderly most likely affected the overall analysis. Evidence suggests that older groups are at higher risk for severe COVID-19 infection; these individuals also have higher perception risks than individuals in younger age groups (29–32). Moreover, there is limited reach of the filled audience because it depends on the initial networks that the researchers deployed, so this cannot be stated as representative. Nevertheless, this research finding may inform and assist the provincial government on evidence-based strategies as it provides self-reported Balinese behaviors, knowledge, and adherence to health protocols. This further indicates the need for better public health measures designed to promote appropriate behaviors during the COVID-19 pandemic. Future studies should therefore consider more representative sampling methods, thus increasing generalizability.

## Conclusions

In Bali Province, several factors influenced the level of adherence to public health measures targeted at COVID-19. More specifically, this included the perception of social norms, perception of health-related benefits and values, gender, and COVID-19 test histories. In the context of COVID-19, such adherence is strongly impacted by the social roles played by individuals within the community, who must make appropriate adaptations to prevent viral transmission. As additional policies should be enacted to reinforce the current level of adaptation, continued research is needed to explore social norms and interactions in the context of the pandemic era. Findings will be crucial, since it is very unlikely that the COVID-19 pandemic constitutes the final global health threat.

## Data Availability Statement

The raw data supporting the conclusions of this article will be made available by the authors, without undue reservation.

## Ethics Statement

The studies involving human participants were reviewed and approved by Ethics Commission, Faculty of Medicine, Udayana University. The patients/participants provided their written informed consent to participate in this study.

## Author Contributions

PI and PJ were responsible for the study conceptualization and supervision and writing the original manuscript draft. PI, PJ, PP, and HG handled the methodological construction/review. PP conducted the formal analysis. PI, PJ, PW, and HG engaged in further writing/review. All authors contributed to the article and approved the submitted version.

## Conflict of Interest

The authors declare that the research was conducted in the absence of any commercial or financial relationships that could be construed as a potential conflict of interest.
