# Supplementary material for: Perceived Social Norms as Determinants of Adherence to Public Health Measures Related to COVID-19 in Bali, Indonesia
Source: Front Public Health. 2021 Apr 30;9:646764. doi: 10.3389/fpubh.2021.646764 (PMC8119776; doi:10.3389/fpubh.2021.646764)
Supplement: Supplementary file 1 [file Data_Sheet_1.docx]

**Supplementary file 1. The Values, Rules, and Knowledge (VRK) Construct Used in This Study**

| **VRK Constructs** |
| --- |
| **Values definition:** perception of the value of public health measures (value of health, business, and social benefits) |
| 1. **Perception of the value of health benefits**   **Definition:** perceived value of health protocol benefits for public health  **Item (3)**   1. The health protocol for wearing a mask during activities has benefited me in maintaining my health 2. The health protocol for washing hands has benefited me in maintaining my health 3. The health protocol for physical distancing has benefited me in maintaining my health |
| 1. **Perception of the value of economic benefits**   **Definition:** perceived value of health protocol benefits for businesses/economies in society  **Item (3)**   1. The health protocol for wearing a mask helps my business and my job 2. The health protocol for washing hands helps my business and my job 3. The health protocol for physical distancing helps my business and my job |
| 1. **Perception of the value of social benefits**   **Definition:** perceived value of health protocol benefits for social life within the community  **Item (1)**  The health protocols for wearing a mask, washing hands, and physical distancing have disturbed my social life |
| **Rules**  **Definition:** rules consist of formal rules and social norms |
| 1. **Social norms**   **Definition:** social norms in society and their enforcement  **Item (6)**   1. People around me reduce gatherings and other social events 2. People in my area keep their distance and reduce physical contact 3. People around me wears masks during activities 4. Those with fever, cough, cold, and/or sore throat avoid crowds 5. People warn others who do not wear masks, wash hands, and/or engage in physical distancing 6. I warn others if they do not wear masks, wash hands, and/or engage in physical distancing |
| 1. **Formal Rules**   **Definition:** government regulations regarding public health measures  **Item (5)**   1. The current regulations for COVID-19 require masks when performing activities outside and working 2. The current regulation for COVID-19 require public facilities to provide areas for washing hands and/or using hand sanitizer 3. The current regulations for COVID-19 may regulate business owners and traders when implementing health protocols 4. The government takes firm actions against violations of public health measures, such as not wearing masks, maintaining physical distance, and washing hands 5. The government takes firm actions to prevent large crowds or gatherings |
| **Knowledge**  **Definition:** knowledge of COVID-19 and public health measures in the era of a new normal  **Item (10)**   1. The main clinical symptoms of COVID-19 are fever, dry cough, sore throat, loss of smell, and breathing difficulties 2. Currently, there is no effective medicine for curing COVID-19; there are only medical treatments for helping patients recover from COVID-19 3. Elderly persons with chronic diseases (hypertension/diabetes) are vulnerable to severe issues with COVID-19 4. People with COVID-19 cannot infect others when there is no fever 5. Transmission occurs due to the inhalation of droplets containing the virus or touching contaminated objects, then touching the face 6. Children and adolescents do not need to implement public health measures (using masks, keeping distance, washing hands) to avoid the COVID-19 virus 7. Community members (non-medical staff) can wear fabric masks to prevent COVID-19 infections 8. To prevent COVID-19 transmission, people should avoid going to crowded places, such as markets and malls 9. Isolating and treating people who are infected with COVID-19 are effective for reducing the spread of the virus 10. People who physically contact others infected with COVID-19 must be properly isolated for 14 days |

**Supplementary file 2. Protocol Adherence**

| **Protocol Adherence inside and outside house** | **Never** | **Rarely** | **Often** | **Always** |
| --- | --- | --- | --- | --- |
| In the past week, I worn have a mask when I get together with my family | 8.95 | 19.61 | 21.97 | 49.47 |
| In the last week, I washed my hands with soap and running water after handling/holds shared things such as gates, TV remote, etc | 0.66 | 6.84 | 31.58 | 60.92 |
| In the last week when I came home, I washed my hands and take off my shoes outside home | 0.26 | 2.37 | 14.47 | 82.89 |
| In the last week, I changed clothes when I got home after activities outside home | 0.13 | 7.11 | 21.84 | 70.92 |
| I cover my nose and mouth when sneezing/coughing at home | 0.66 | 3.55 | 20.13 | 75.66 |
| In the last week, I used mask while outside home | 0 | 1.21 | 10.77 | 88.02 |
| In the last week, I keep distance 1-2 meter when chatting with others outside home | 0.81 | 9.29 | 31.22 | 58.68 |
| I cover my nose and mouth when sneezing/coughing outside home | 0.27 | 0.67 | 11.31 | 87.75 |
| In the last week, I avoided crowded | 0.13 | 4.17 | 32.57 | 63.12 |

**Supplementary file 3. Perception of the value of value of health, business, and social benefits**

| **Perception of the value of value of health, business, and social benefits** | **Strongly**  **Disagreed** | **Disagreed** | **Agreed** | **Strongly Agreed** |
| --- | --- | --- | --- | --- |
| The health protocol wearing mask in activities, has benefits in maintaining my health | 0 | 1.32 | 39.08 | 59.61 |
| The health protocol washing hands in activities, has benefits in maintaining my health | 0 | 0.13 | 31.71 | 68.16 |
| The health protocol keeping physical distance in activities, has benefits in maintaining my health | 0 | 0.92 | 42.63 | 56.45 |
| The health protocol washing hands has benefits in securing my business/job | 0.13 | 0.26 | 40.92 | 58.68 |
| The health protocol wearing mask has benefits in maintaining my business/job | 0 | 1.97 | 42.11 | 55.92 |
| The health protocol keeping physical distance has benefits in maintaining my business/job | 0.13 | 2.76 | 43.95 | 53.16 |
| Health protocol wearing masks, washing hands, keeping physical distance disrupt my social life | 13.29 | 57.5 | 17.63 | 11.58 |

**Supplementary file 4. Perception of Rule**

| **Social Norms** | **Never** | **Rarely** | **Often** | **Always** |
| --- | --- | --- | --- | --- |
| People around me reduces gathering and social events | 1.45 | 18.55 | 45.92 | 34.08 |
| People in my area keep their distance and reduce physical contacts | 1.05 | 21.05 | 47.5 | 30.39 |
| People around me wear masks during their activities | 0.39 | 7.89 | 39.87 | 51.84 |
| Those with fever, cough, cold, and sore throat avoids crowd | 2.5 | 11.58 | 41.97 | 43.95 |
| People warned others if not wearing mask, keeping physical distance, and washing hands | 3.95 | 22.89 | 39.74 | 33.42 |
| I warned others if not wearing mask, keeping physical distance, and washing hands | 3.82 | 17.24 | 38.42 | 40.53 |

| **Formal Rules** | **Strongly**  **Disagreed** | **Disagreed** | **Agreed** | **Strongly Agreed** |
| --- | --- | --- | --- | --- |
| Current regulation regarding COVID-19 make people wear masks while doing activities and working outside home | 0.39 | 1.45 | 45.92 | 52.24 |
| Current regulation regarding COVID-19 make public facilities provide a place for washing hand and/or hand sanitizer | 0.13 | 0.79 | 41.84 | 57.24 |
| Current regulation regarding COVID-19 able to regulate businessman and traders to implement health protocol | 0.53 | 3.95 | 49.34 | 46.18 |
| The government has taken strict action againts violations of health protocol | 2.76 | 15.66 | 45.26 | 36.32 |
| The government has act decisively to prevent large crowd | 2.37 | 12.24 | 45.79 | 39.61 |

**Supplementary file 5. Knowledge**

|  | **True** | **False** | **Don't know** |
| --- | --- | --- | --- |
| Main clinical symptom of COVID-19 are fever, dry cough, sore throat, loss of smell to breath difficulties | 93.16 | 0.79 | 6.05 |
| Currently there is no effective medicine to cure COVID-19, only medical treatment to help patients recover from COVID-19 | 88.55 | 1.71 | 9.74 |
| Elderly with chronic disease (hypertension/diabetes) are vulnerable to be severe due to COVID-19 | 93.68 | 0.26 | 6.05 |
| People with COVID-19 cannot infect others when there is no fever | 10.79 | 74.47 | 14.74 |
| Transmission occurs due to inhalation of droplets containing viruses or touching contaminated objects then the hands touching face | 95.39 | 0.53 | 4.08 |
| Children and adolescents do not need to implement public health measures (using masks, keeping distance, washing hands) to avoid COVID-19 | 6.58 | 90.79 | 2.63 |
| Community (non-medical staff) can wear fabric mask to prevent COVID-19 | 91.84 | 3.29 | 4.87 |
| To prevent COVID-19 transmission, people should avoid going to crowded places such as market, malls, etc | 94.08 | 3.55 | 2.37 |
| Isolation and treatment of people infected with COVID-19 is an effective way to reduce the spread of the virus | 96.58 | 0.53 | 2.89 |
| People who have contact with someone infected with COVID-19 must be properly isolated for 14 days | 96.32 | 0.79 | 2.89 |

**Supplementary file 6. Perception of fear**

| **Perception of fear** | **Strongly Disagreed** | **Disagreed** | **Neutral** | **Agreed** | **Strongly Agreed** |
| --- | --- | --- | --- | --- | --- |
| I am afraid of COVID-19 | 3.42 | 12.89 | 27.37 | 40.39 | 15.92 |
| I am afraid losing my life due to COVID-19 | 4.34 | 10.79 | 19.87 | 42.11 | 22.89 |
| When thinking of COVID-19, I feel uncomfortable | 3.95 | 15.26 | 22.11 | 45.26 | 13.42 |
| My hand getting wet when thinking of COVID-19 | 23.95 | 51.58 | 16.84 | 6.05 | 1.58 |
| I could not sleep because I was worry about COVID-19 | 24.47 | 47.24 | 19.47 | 6.71 | 2.11 |
| When I observe all information (watching news and stories) about COVID-19 on social media, I became nervous and anxious | 10.53 | 29.08 | 32.5 | 23.82 | 4.08 |
| I feel my heart beating fast if I think I have COVID-19 | 14.08 | 31.97 | 24.87 | 24.74 | 4.34 |

**Supplementary file 7. Access to COVID-19 prevention instruments**

| **Access to COVID-19 prevention instruments** | **Strongly Disagreed** | **Disagreed** | **Agreed** | **Strongly Agreed** |
| --- | --- | --- | --- | --- |
| I can easily get a mask | 0.53 | 10.53 | 55.26 | 33.68 |
| I can easily find a place to wash my hands with soap and water | 0.26 | 6.84 | 60.79 | 31.11 |
| I get used to wash my hands with soap and running water | 0 | 1.05 | 46.32 | 52.63 |
| I can easily get a hand sanitizer | 1.45 | 10 | 57.63 | 30.92 |
